# Antifungal Activity of Mycogenic Silver Nanoparticles on Clinical Yeasts and Phytopathogens

**DOI:** 10.3390/antibiotics12010091

**Published:** 2023-01-05

**Authors:** Luiz Gustavo Ribeiro, Gabriella Sales Calaço Roque, Rafael Conrado, Ana Olívia De Souza

**Affiliations:** 1 Development and Innovation Laboratory, Instituto Butantan, Avenida Vital Brasil, 1500, São Paulo 05503-900, SP, Brazil; 2 Department of Surgery, Faculty of Veterinary Medicine and Animal Science, University of São Paulo, São Paulo 05508-270, SP, Brazil

**Keywords:** biogenic silver nanoparticles, phytopathogens, *Candida* sp., antifungal activity

## Abstract

In this study, seven different silver nanoparticles (AgNPs) were obtained using the fungi species from the phylum Ascomycota, *Aspergillus tubingensis*, *Aspergillus* spp., *Cladosporium pini-ponderosae*, *Fusarium proliferatum*, *Epicoccum nigrum*, *Exserohilum rostratum*, and *Bionectria ochroleuca*, isolated from the Brazilian biodiversity, particularly from the mangrove and Caatinga biomes. The nanoparticles were coded as AgNP-AT, AgNP-Asp, AgNP-CPP, AgNP-FP, AgNP-EN, AgNP-ER, and AgNP-BO and characterized using spectrophotometry (UV-Vis), dynamic light scattering (DLS), zeta potential, transmission electron microcopy (TEM), and Fourier-transform infrared (FTIR) spectroscopy. All the AgNPs presented homogeneous size in the range from 43.4 to 120.6 nm (DLS) and from 21.8 to 35.8 nm (TEM), pH from 4.5 to 7.5, negative charge, and presence of protein coating on their surface. The antifungal activity of the AgNPs was evaluated on clinical strains of *Candida albicans*, and on the non-albicans species, *Candida krusei*, *Candida glabrata*, *Candida parapsilosis*, *Candida tropicalis*, and *Candida guilliermondii*, common in hospital infections, and against the phytopathogens *Fusarium oxysporum, Fusarium phaseoli*, *Fusarium sacchari*, *Fusarium subglutinans*, *Fusarium verticillioides*, and *Curvularia lunata*, which are species responsible for serious damage to agriculture production. The AgNPs were effective against the yeasts with MICs ranging from 1.25 to 40 µM and on the phytopathogens with MICs from 4 to 250 µM, indicating the promising possibility of application of these AgNPs as antifungal agents. The results indicated that the physicochemical parameters of the AgNPs, including the functional groups present on their surface, interfered with their antifungal activity. Overall, the results indicate that there is no specificity of the AgNPs for the yeasts or for the phytopathogens, which can be an advantage, increasing the possibility of application in different areas.

## 1. Introduction

The growth of fungal infections has become a major threat to public health, with more than 300 million people suffering from fungal diseases each year, resulting in over 1 million deaths worldwide [1,2]. In addition to infections caused by fungi in humans and animals, there are also problems caused by phytopathogens in several types of crops that are important for food production [3,4].

A recent study reported that at least 137 phytopathogens and pests are responsible for infections in potato, wheat, rice, maize, and soybean, causing massive crop losses [5]. The loss in the production is estimated to be 17.2% for potato, around 20% for soybean, wheat, and maize, and 30.0% for rice [5]. The food loss is important in all countries; however, the maximum loss has been observed in food-insecure regions with high populations, significantly increasing the problem of starvation [5].

The situation is worsened by the increased resistance of these pathogens to the currently available treatments, which are scarce, for infections both in humans [2] and animals [6] and for phytopathogens [7]. Microbial resistance is considered a global health problem, which compromises the effectiveness of antibiotics, making the treatment of common infections unfeasible [8,9].

*Candida* sp. species are a global threat to public health, with frequent outbreaks in hospitals and a high rate of mortality [10]. The genus Candida can be found in several ecosystems, such as in the microbiota of man and of some animals. *Candida* sp. species degrade proteins and carbohydrates to acquire carbon and nitrogen for their development and have an adaptive ability to multiply in aerobic and anaerobic conditions. These species are commensal and usually are present in the gastrointestinal tract, vagina, urethra, and lung microbiota. In case of an imbalance in the body’s microbiota, *Candida* sp. can become pathogenic for the host, causing candidiasis or even a severe candidemia, being, due to that, considered opportunistic microorganisms [11,12,13,14].

Among 15 Candida species usually present in infections in humans, *Candida albicans*, *Candida glabrata*, *Candida tropicalis*, *Candida parapsilosis*, and *Candida krusei* are the most common, causing more than 90% of all the candidemia cases [15].

A study performed in Portugal, in 2016 and 2017, reported 117 cases of candidemia in 114 patients, and among them, 51.3% were caused by *C. albicans*, followed by 22.2%, 15.4%, 4.3%, and 2.6% caused by *C. glabrata*, *C. parapsilosis*, *C. tropicalis*, and *C. lusitaniae*, respectively [10].

The most common fungi species responsible for frequent and problematic contamination of foods and feeds due to mycotoxins production belongs to the genera *Aspergillus*, *Fusarium*, and *Penicillium* [16,17,18]. Under natural conditions, these species are able to produce mycotoxins, in pre- and post-harvest cultures, which can be extremely harmful to humans and animals. The exposure to these mycotoxins occurs primarily by ingestion but can also occur by the dermal and inhalation routes. The effects of some food-borne mycotoxins can be acute, with symptoms of severe illness appearing rapidly after the consumption of food products contaminated [16,17,18,19].

For the treatment and successful control of the serious threats caused by phytopathogenic and human pathogenic fungi, new strategies against the shortage of antifungal drugs are necessary.

Nanomaterials of sizes between 1 and 100 nm have been successfully used in different medical and pharmaceutical applications [20]. In general, nanoscale materials have excellent antimicrobial activity due to the high surface area to volume ratio and physical chemical properties [20]. Several studies have demonstrated the antifungal activity of metallic nanoparticles, and among them, silver nanoparticles (AgNPs) represent a potential option as antimicrobial agents [20,21,22,23,24,25].

AgNPs can be obtained using physical, chemical, or biological methods [20,23,24,25]. However, biological synthesis stands out as an ecologically sustainable process, economically viable and able to be scaled up to industrial production without a big challenge. In the biological route, factors such as pH, luminosity, chemical characteristics, and concentration of the substrate or reducing agents are factors that can interfere with the nucleation, growth, agglomeration, and stability of the nanoparticles [22,23,25].

Through the bioreduction of the metal precursor (i.e., silver, gold), reducing or stabilizing agents produced by plants, algae, bacteria, yeasts, or fungi, such as ether, thiol, carbonyl, hydroxyl, amine, polyamine, proteins, and peptides groups, can also perform a coat on the metallic nanoparticles surface [22,23,25]. These biological coating groups, usually from proteins [26], can improve the uptake and functionality of the nanoparticles, reducing their toxicity and improving their potential as candidates to treat diseases in humans, animals, and in agriculture [20,21,27,28].

The mycogenic biosynthesis of AgNPs has been reported with the application of several species of fungi [22,23,25,29] and presence of a protein coating surrounding the metal ion, which provides high stability, avoiding aggregation of the nanoparticles [22,23,25]. This property is an advantage that improves the interaction of the nanoparticles with biological targets and their biological effect [27,28].

Among the possible organisms capable of biosynthesizing AgNPs, fungi offer advantages because they are easy to manipulate, allowing a large-scale culture and biomass production that can be used for metal nanoparticles biosynthesis, which own a protein coat that can contribute to an improved biological activity [22,23,25].

Considering the high demand for new antifungal agents, due to the emergence of microbial resistance to the existing treatments and the potential of AgNPs as an antifungal alternative, in this study seven AgNPs were obtained by green synthesis, using seven species of fungi isolated from the Brazilian biodiversity, particularly from the mangrove and Caatinga biomes. The mangroves in the Brazilian coast are anaerobic and saline, and the Caatinga is an arid environment with high temperature and hot soil through all the year. Both these environments harbor extremophiles microorganisms, and the fungi *Aspergillus* spp., *Cladosporium pini-ponderosae*, *Fusarium proliferatum*, *Epicoccum nigrum*, *Aspergillus tubingensis*, and *Bionectria ochroleuca* applied in this study were previously isolated from mangrove, while *Exserohilum rostratum* was isolated from the Caatinga biome.

To the best of our knowledge, this is the first report on the biosynthesis of AgNPs using the species of *F. proliferatum*, *C. pini-ponderosae*, and *E. rostratum*. The obtained AgNPs were characterized according to their physicochemical properties, using spectrophotometry (UV-Vis), Fourier-transform infrared (FTIR) spectroscopy, transmission electron microscopy (TEM), dynamic light scattering (DLS), and zeta potential. The antifungal activity of the AgNPs was evaluated in *C. albicans*, *C. krusei*, *C. glabrata*, *C. parapsilosis*, *C. tropicalis*, and *Candida guilliermondii*, that are important pathogenic yeasts present in hospital infection [10], and on the phytopathogens *Fusarium oxysporum*, *Fusarium phaseoli*, *Fusarium sacchari*, *Fusarium subglutinans*, *Fusarium verticillioides*, and *Curvularia lunata*, which are species responsible for serious damage in the agricultural production.

## 2. Results and Discussion

### 2.1. Physicochemical Characterization of the AgNPs

The AgNPs obtained using the fungi *Aspergillus* spp., *E. nigrum*, *F. proliferatum*, *C. pini-ponderosae*, *E. rostratum*, *A. tubingensis*, and *B. ochroleuca* were coded by the initial of the respective fungus species applied in their biosynthesis, being AgNP-Asp, AgNP-EN, AgNP-FP, AgNP-CPP, AgNP-ER, AgNP-AT, and AgNP-BO, respectively.

**Figure 1:**
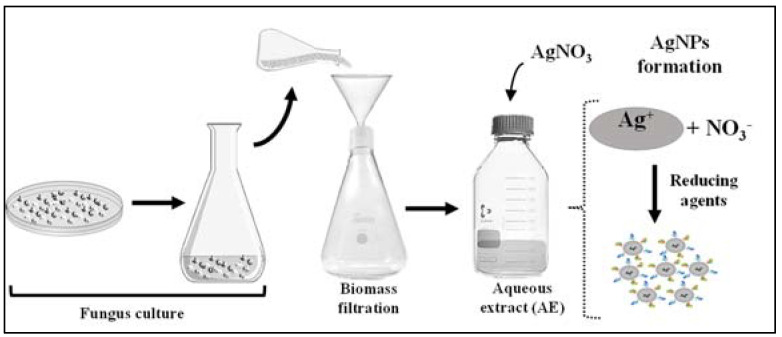
Representation of the silver nanoparticles biosynthesis by the reaction of the extracellular cell free aqueous extract (AE) obtained from the fungi culture with AgNO_3_.

**Figure 2:**
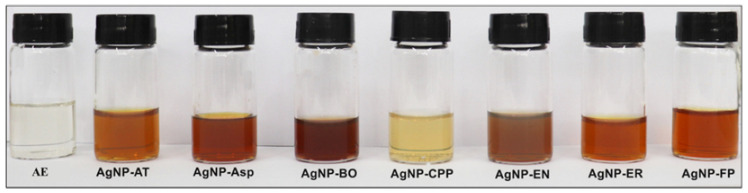
Color of the AgNP-AT, AgNP-Asp, AgNP-BO, AgNP-CPP, AgNP-EN, AgNP-ER, and AgNP-FP formed by the reaction of the extracellular cell free aqueous extract (AE) obtained using the fungi *A. tubingensis*, *Aspergillus* spp., *B. ochroleuca*, *C. pini- ponderosae*, *E. nigrum*, *E. rostratum*, and *F. proliferatum* with AgNO_3_. Pictures were obtained 96 h after the beginning of the reaction.

The formation of the AgNPs was observed by the reaction of the extracellular cell free aqueous extract (AE) obtained from the culture of the seven fungi species with AgNO_3_ as represented in Figure 1. As can be observed in Figure 2, there was a color change of the reactional mixtures from colorless to brownish, indicating the formation of AgNPs in the first hours of the reaction.

**Figure 3:**
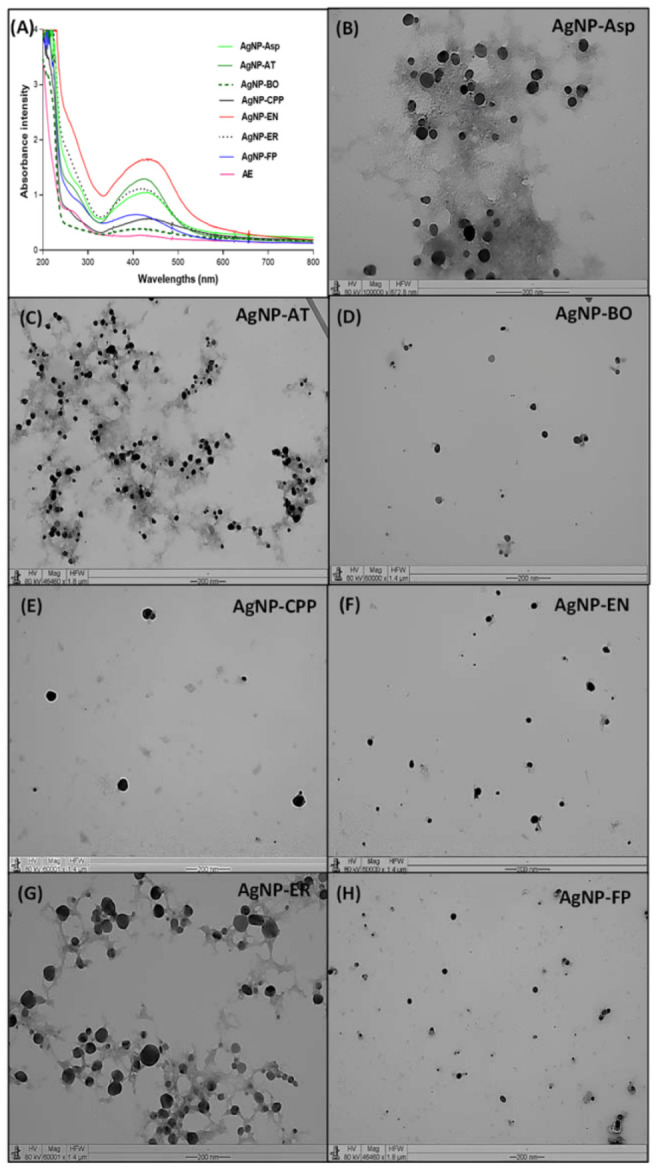
AgNPs characterization by (**A**) UV-Vis profile from 200 to 800 nm with presence of surface plasmon resonance around 420 nm for the AgNP-Asp, AgNP-AT, AgNP-BO, AgNP-CCP, AgNP-EN, AgNP-ER, and AgNP-FP, obtained using the extracellular cell free aqueous extract (AE) of the culture of *Aspergillus* spp., *A. tubingensis*, *B. ochroleuca*, *C. pini-ponderosae*, *E. nigrum*, *E. rostratum*, and *F. proliferatum*, respectively. Transmission electron microscopy for (**B**) AgNP-Asp (magnification 100,000×), (**C**) AgNP-AT (magnification 46,640×), (**D**) AgNP-BO (magnification 60,000×), (**E**) AgNP-CPP (magnification 60,001×), (F) AgNP-EN (magnification 50,000×), (**G**) AgNP-ER (magnification 60,001×), and (**H**) AgNP-FP (magnification 46,640×). The extracellular cell free aqueous extracts (AE) obtained from all fungi culture were used as control for the UV-Vis analysis and showed the same profile.

The resonant oscillation of electrons density present on the surface of the AgNPs resulted in the formation of the surface plasmon resonance observed at 420 nm for all AgNPs (Figure 3A), which is the main characteristic of AgNPs formation [30].

It is possible to observe a difference in the intensity of the brownish color of the AgNPs (Figure 2) and in the absorbance (Figure 3A), which was higher for the AgNP-EN, followed by AgNP-ER, AgNP-Asp, AgNP-FP, and AgNP-CPP. The color intensity is related to the concentration of AgNPs formed after 96 h of reaction. The data allow inferring that the AgNP-EN was formed faster than the other AgNPs. The hydrodynamic sizes of the AgNPs measured by DLS were 44.9 ± 4.1, 43.4 ± 3.3, 120.6 ± 3.5, 87.1 ± 3.4, 71.2 ± 6.7, 86.4 ± 6.4, 59.6 ± 2.1 nm for the AgNP-Asp, AgNP-AT, AgNP-BO, AgNP-CPP, AgNP-EN, AgNP-ER, and AgNP-FP, respectively (Table 1). The sizes of the AgNPs measured by TEM were, respectively, 33.3 ± 2.7, 25.0 ± 6.5, 21.8 ± 4.1, 35.8 ± 5.16, 28.0 ± 6.31, 22.1 ± 2.9, 26.7 ± 5.3 nm. As expected, the sizes measured by TEM were smaller than those measured by DLS. Through the DLS, the protein corona surrounding the metal ion in the nanoparticles is also measured, and consequently, the size of the nanoparticles is higher. This data is interesting since the protein corona can interfere with the interaction of the nanoparticles with the target [27,28].

The polydispersity index (PDI) is related to the distribution of the AgNPs sizes. The smaller the PI values, the more homogeneous is the size of the AgNPs. The AgNP-AT presented the lowest PI, followed by AgNP-FP, AgNP-ER, AgNP-BO, AgNP-CPP, AgNP-Asp, and AgNP-EN. These data can be confirmed by the size and morphology observed for each AgNPs through the analysis by using TEM (Figure 3B–H). It can be also observed that all AgNPs are predominantly spherical, with a few or any aggregate formations (Figure 3B–F).

Literature describes that smaller AgNPs can be more cytotoxic than larger ones, due to intracellular uptake and greater surface area, which can promote more effective interaction with cells or their intracellular components [27,28,31]. The surface charge of AgNPs is defined by the presence of negatively or positively charged molecules on their coat, which is a relevant property for the nanoparticles’ stability. The negative charge generates repulsiveness between particles and a low constant of force between then, preventing the formation of aggregates [32]. All seven AgNPs obtained in this study showed negative zeta potential values (Table 1) and, as it is shown in Figure 3B–F, there was no aggregation formation for almost all the AgNPs.

The pH value is an interesting parameter due to its influence on the size, shape, dispersion, and protein coating of AgNPs [33]. In the present study, the AgNPs with larger sizes showed lower pH values. Similar results were previously demonstrated in a study performed by Prakash and Soni [33], in which the size and shape of the AgNPs obtained using the fungi *Chrysosporium tropicum* and *F. oxysporum* were according to the pH and temperature applied for the synthesis. The pH of biogenic AgNPs is related to its protein coat, and both can interfere with the nanoparticles function and activity in biological systems, according to the possibility of nano–bio interaction [27,28,31].

**Figure 4:**
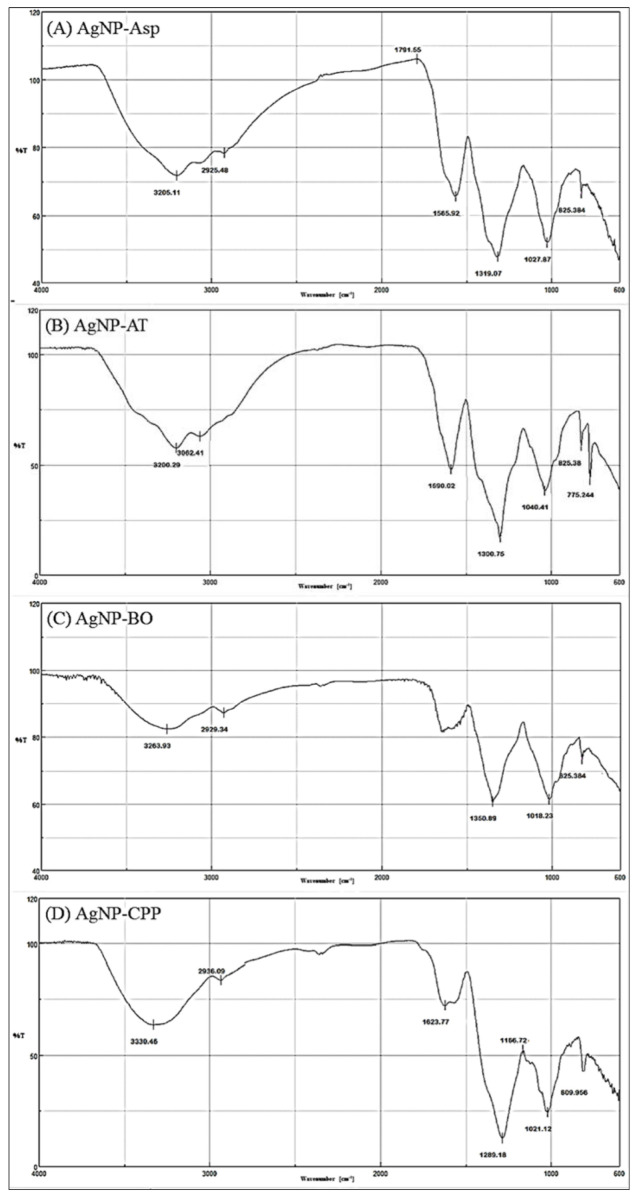
Fourier-transform infrared (FTIR) spectroscopy profile for the (**A**) AgNP-Asp, (**B**) AgNP-AT (**C**) AgNP-BO, and (**D**) AgNP-CPP.

**Figure 5:**
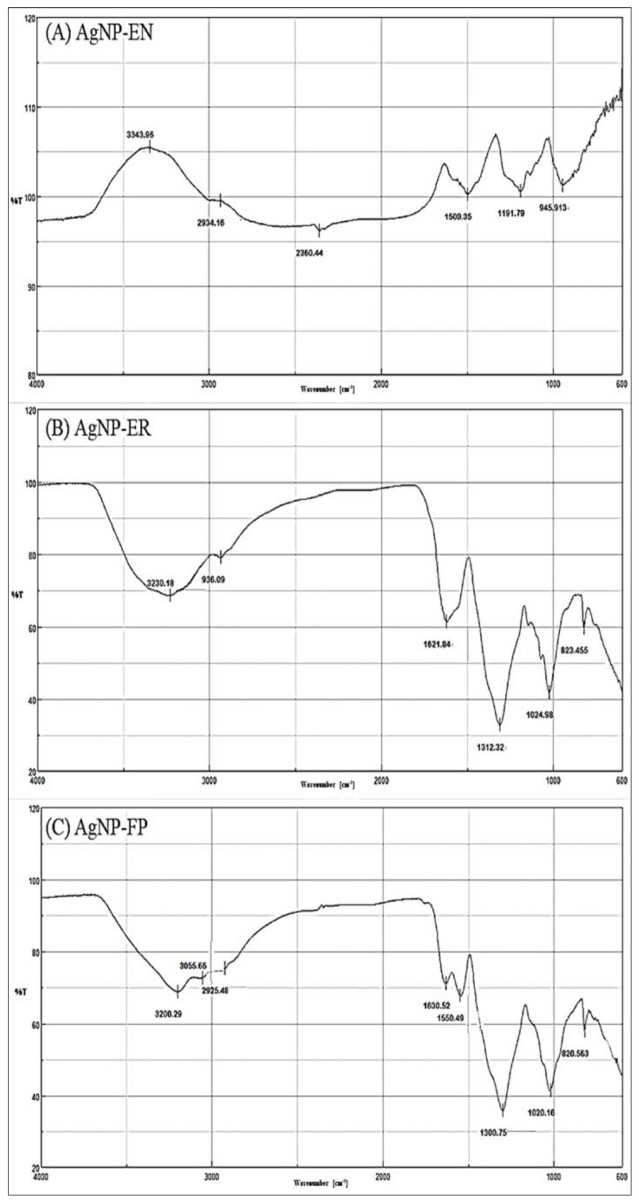
Fourier-transform infrared (FTIR) spectroscopy profile for the (**A**) AgNP-EN, (**B**) AgNP-ER, and (**C**) AgNP-FP.

The FTIR spectroscopy of the AgNPs showed the presence of functional groups attributed to aromatic rings, ether, carboxylic, and amide groups in their composition, which are from molecules present in the AE. These molecules acted as reducing agents for the formation of the AgNPs, or as capping agents promoting the protein corona formation and stability of the AgNPs (Table 2 and Figure 4 and Figure 5). Additional functional groups were detected and attributed to amino acid residues present in the side chains of proteins presents in the AE (Table 2, and Figure 4 and Figure 5).

The functional groups identified by FTIR for AgNP-Asp, AgNP-CCP, AgNP-EN, AgNP-ER, and AgNP-FP, and shown in Table 2, indicate the presence of several molecules around the nanoparticles’ surface, as those previously reported for AgNP-AT and AgNP-BO [26,34]. The presence of a band in the region of 3200 cm^−1^ for all AgNPs was attributed to OH (chelate) groups and indicate the chelation of the metal ion (Ag^+^) by molecules from the AE used for the biosynthesis of the AgNPs (Table 2 and Figure 4 and Figure 5), as it was also observed for the AgNPs obtained using other microorganisms [35]. The results indicate that the secondary structure of proteins present in the AE used for the AgNPs biosynthesis was not modified by the oxidation–reduction reaction, or binding to the metallic core [26,34].

The effectiveness of AgNPs against specific targets is related to several aspects such as the morphology, size, PI, zeta potential, and pH values. In general, the AgNPs obtained in this study showed different functional groups on their surface forming the protein corona and interesting physicochemical properties, which are suitable for antimicrobial application. These groups were identified by FTIR spectroscopy and come from the AE used for the biosynthesis of the AgNPs (Table 2 and Figure 4 and Figure 5). It is known that small and homogeneous size and absence of aggregates are interesting for the interaction of nanoparticles with biological systems [27,28,31], and in this study, in general the AgNPs showed this profile in their properties.

### 2.2. Antifungal Activity

AgNPs have a broad spectrum of antimicrobial activity [20,21,22,23,24,34,35,36,37], including the ability to inhibit biofilm formation [36], and, due to that, are a promising option as antifungal agents. In this study, the antifungal activity of the AgNPs was evaluated against different species of *Candida* sp., common in hospital infections and against different relevant phytopathogens, capable of causing agricultural damage. The AgNPs showed antifungal activity against clinical strains of *C. albicans*, *C. krusei*, *C. glabrata*, *C. guillermondii*, *C. parapsilosis*, and *C. tropicalis*. The AgNPs were more effective than amphotericin B (AMB), which was used as the positive control, and was not effective until 32 µM in all the *Candida* sp. species. The AgNPs showed MIC in the range from 1.25 to 40 µM, and *C. guillermondii*, *C. krusei*, and *C. glabrata* were the most sensitive to the action of all AgNPs that showed MIC from 1.25 to 5 µM (Table 3). The only exception was the AgNP-FP, that until 40 µM was not effective on *C. glabrata*. It is noteworthy that the AgNP-CPP was not effective on any of the four *C. albicans* strains evaluated, but it was effective on non-albicans species with MICs of 1.25 to 5 µM (Table 3).

The AgNPs were effective on *C. parapsilosis* and *C. tropicalis*, but with MIC from 2.5 to 40 µM. Until 40 µM, the AgNP-Asp was not effective on *C. tropicalis* and AgNP-FP on *C. parapsilosis*.

The AgNPs showed antifungal activity against the phytopathogens *C. lunata*, *F. subglutinans*, *F. verticillioides*, and on one strain of *F. oxysporum*. The AgNPs showed higher MIC on the *F. oxysporum* WLA-FP07 that was less susceptible to their activity. These phytopathogens cause diseases in sugarcane and in crops such as in rice, maize, and beans with a great impact on agriculture and economy [38,39,40]. Synthetic fungicides are used in the management of these diseases, and their systematic and uncontrolled use has often led to fungal resistance [41].

Among the seven AgNPs, until 250 µM, none prevented the growth of *F. sacchari* (Table 4). The phytopathogens *C. lunata* and *F. verticillioides* were the most susceptible to the AgNPs, and even at low concentrations, all the AgNPs prevented the growth of these fungi (Table 4). These phytopathogens cause diseases in different cultivar species, and their impact on the economy is very relevant [42].

On the phytopathogens, the AgNPs showed the same values for MICs and fungicide concentration (FC), indicating that the activity of AgNPs is more fungicide than fungistatic.

In this study, the AgNPs presented sizes in the range from 43.4 to 120.6 nm (DLS) and from 21.8 to 35.8 nm (TEM), spherical and regular forms, with negative zeta potential and pH ranging from slightly acidic to neutral. The participation of biological molecules in the biosynthesis of AgNPs confers interesting advantages to the AgNPs, improving the interaction with biological targets and the biocompatibility [27,28,31]. The influence of these factors can be related to the antifungal activity.

The antifungal activity of biological AgNPs was previously described by several authors [20,21,22,23,24,34,35,36,37,43]. In the current study, the higher antifungal activity was observed for the AgNP-Asp on the different Candida species, including the non-albicans. However, the AgNP-CPP was the less effective.

An analysis of the physicochemical parameters (Table 3) for the AgNP-Asp and AgNP-CPP indicates that the size was important for the antifungal activity. The first one has a size of 44.9 and the last one of 87.1 nm. The lower size contributes to a better activity. Between both nanoparticles, there was not a high difference in pH, zeta potential, and PDI. On the yeasts, the range of MICs values obtained for the different AgNPs was from 1.25 to 40 μM. The MICs were different on the different strains of *C. albicans*, and that from ATCC was more susceptible to the action of AgNPs, while clinical isolates were more resistant. These differences were expected due to a possible genetic variability, and therefore it is important to test the susceptibility of different strains, especially of clinical isolates to the AgNPs activity.

The FTIR analysis showed the presence of aliphatic (C-H) functional group for AgNP-Asp, AgNP-AT, AgNP-BO, AgNP-ER, and AgNP-FP, and these nanoparticles were the most effective in killing the phytopathogens and yeasts, with lower MICs values. Interestingly, the AgNP-CPP and AgNP-EN did not present this functional group and were the less effective AgNPs on the yeasts and on phytopathogens with higher MICs value. This functional group has already been described as a potent antifungal agent against *Saccharomyces cerevisiae*, where primary aliphatic alkanols were tested and showed minimum fungicidal concentration (MFC) of 25 μg/mL (0.14 mM) [44].

The comparison between the physicochemical parameters of the AgNP-Asp and AgNP-FP shows a difference in their size and zeta potential. AgNP-FP has size and zeta potential of 56.9 ± 2.1 nm and −15.33, while for the AgNP-Asp these values are 44.9 ± 4.1 nm, and −1.04. Both nanoparticles have pH of 6.0 and are surrounded by molecules containing the functional aliphatic group. The AgNP-Asp is smaller with higher zeta potential value and was more effective then AgNP-FP as an antifungal agent on the yeasts. As previously reported [27,28,31], these data indicate that all the parameters are related and can interfere with the antifungal activity.

The results showed that the AgNPs were very effective against clinical yeasts and phytopathogens. The AgNPs were effective against phytopathogens, showing MICs from 4 to 120 µM, and against yeasts with MICs range of 1.25 to 40 µM, indicating the promising possibility of application of these AgNPs as an antifungal agent.

## 3. Materials and Methods

### 3.1. Fungal Strains

Fungi species employed in the AgNPs biosynthesis were previously isolated from the Brazilian biodiversity and deposited at the microorganisms collection from Instituto Oswaldo Cruz (IOC, Rio de Janeiro, RJ, Brazil), Instituto Adolfo Lutz (IAL, São Paulo, SP, Brazil), or in the “Collection of Microorganisms for Biocontrol of Phytopathogens and Weeds” at Embrapa Genetic Resources and Biotechnology (CENARGEN, Brasília, DF, Brazil). The species *Aspergillus* spp. is an endophyte and *C. pini-ponderosae* (IAL 7248), *F. proliferatum* (IOC 4682/IAL 7246), and *E. nigrum* (IAL 7249) are epiphytes from the leaf of the mangrove plant *Rhizophora mangle*, respectively. The *E. rostratum* (IAL 7247) was isolated as an endophyte from the plant *Croton blanchetianus*, collected in the Brazilian biome called Caatinga.

The pathogen *C. albicans* (ATCC 36802/IOC 3704) is a strain from the American Type Culture Collection (ATCC), and *C. albicans* (IOC 4525, IOC 4556, IOC 4558), *C. krusei* (IOC 4559), *C. glabrata* (IOC 4565), *C. parapsilosis* (IOC 4564), *C. tropicalis* (IOC 4560), and *C. guilliermondii* (IOC 4557) are clinical species from the IOC collection. The Fusarium species were isolated from the *Rhizosphere* of sugar cane [45] by the group of Prof. Welington L. Araújo, from Institute of Biomedical Sciences of the University of São Paulo, that kindly provided the phytopathogens from his collection (WLA) for this study. The phytopathogens were *C. lunata* (WLA-FP06), *F. sacchari* (WLA-FP04), *F. subglutinans* (WLA-FP21), *F. oxysporum* (WLA-FP07), *F. oxysporum* (WLA-FP25), and *F. verticillioides* (WLA-FP05).

### 3.2. Biosynthesis and Physicochemical Analysis of the AgNPs

The biosynthesis of the AgNPs was performed according to the protocol previously described by our group [34]. The fungi *A. tubingensis*, *Aspergillus* spp., *B. ochroleuca*, *C. pini-ponderosae*, *F. proliferatum*, *E. nigrum*, and *E. rostratum* were cultivated in potato dextrose agar (PDA, Himedia #M096) for a week at 28 °C and sub-cultivated in an Erlenmeyer flask of 500 mL with 150 mL of potato dextrose broth (PDB) (Himedia #M096) for 72 h at 28 °C and 150 rpm, in an orbital shaker (Marconi MA-420, Piracicaba, SP, Brazil). After filtration through a polypropylene membrane, the biomass was washed with sterile deionized water to remove any residue of the culture medium. The biomass was incubated with sterile deionized water (10 g/100 mL) at 28 °C and 150 rpm for 72 h. Subsequently, following a filtration and biomass discard, the supernatant was sterilized using a 0.22 μm polyethersulfone (PES) membrane, resulting in the aqueous extracellular cell free extract (AE). For the AgNPs biosynthesis, silver nitrate (AgNO_3_) was added to the sterile AE for the final concentration of 1 mM. The reactional mixture was protected from light, and the formation of AgNPs was monitored by reading the absorbance from 200 to 800 nm (UV-Vis, Agilent 8453), showing the presence of a surface plasmon resonance around 420 nm.

The obtained AgNPs were coded according to the initials of the fungi species used for their biosynthesis being AgNP-AT, AgNP-Asp, AgNP-BO, AgNP-CPP, AgNP-FP, AgNP-EN, and AgNP-ER. The AgNPs were characterized by size (Dynamic Light Scattering, DLS, Nanoplus–Particulate Systems, Norcross, GA, USA), polydispersity index (PDI), zeta potential, pH, Fourier-transform infrared (FTIR) spectroscopy, and morphology by transmission electron microscopy (TEM, Zeiss LEO 906 E, de 120Kv, Freiburg, Germany), as previously described [26,34]. The size of the AgNPs was also measured by TEM for at least seven particles of each AgNPs and expressed by the mean ± the standard deviation.

### 3.3. Antifungal Activity Assay

The antifungal activity was evaluated on four strains of *C. albicans* (ATCC 36802/IOC 3704, IOC 4525, IOC 4556, IOC 4558), *C. krusei* (IOC 4559)*, C. glabrata* (IOC 4565), *C. parapsilosis* (IOC 4564), *C. tropicalis* (IOC 4560), and *C. guilliermondii* (IOC 4557). The phytopathogens utilized were *C. Lunata*, *F. phaseoli*, *F. sacchari*, *F. subglutinans*, *F. oxysporum* (WLA-FP07 and WLA-FP25), and *F. verticillioides*.

The yeasts and phytopathogens were cultured on PDA and sub-cultured in PDB. The yeast suspensions were prepared in RPMI 1640 (Gibco #23400-013) culture media at 0.5–2.5 × 10^3^ CFU/mL, according to standard curves previously established in our laboratory. For the phytopathogens, the spores were prepared in a saline solution at 0.9% and after being counted using a Neubauer chamber, the suspensions were prepared in RPMI 1640 with 1 × 10^6^ spores/mL.

The antifungal assay was evaluated by the microdilution assay in a 96-well plate [46,47,48]. The AgNP-AT, AgNP-Asp, AgNP-BO, AgNP-CPP, AgNP-EN, AgNP-ER, and AgNP-FP were serially diluted in RPMI 1640, and each well received 100 µL of the yeasts or phytopathogens suspensions and 25 µL of resazurin dye at 0.02% in saline solution. The AgNPs were assayed at final concentrations of 1.25, 2.5, 5, 10, 20, and 40 μM for the yeasts, and of 4, 8, 16, 30, 60, 120, and 250 μM for the phytopathogens. Untreated culture that received only RPMI 1640 was used as the negative control and amphotericin B (AMB) at 16 and 32 μM was used as positive control. The plates were incubated at 30 °C for 24 h, and the minimal inhibitory concentration (MIC90) was defined as the lowest concentration in which the color of the dye resazurin was kept in blue due to the inhibition of at least 90% of the microorganism’s growth [46]. For the yeasts, the MICs were expressed by the mean of three independent experiments, in duplicate for each AgNPs concentration, and for the phytopathogens by the mean of two assays in triplicate. The fungicidal concentration (FC) for phytopathogens was determined from the MICs. For that, 50 μL of two concentrations before and after the MICs were collected from the plate and incubated in Petri dishes containing PDA at 30 °C for 120 h. The absence of phytopathogens growth was defined as the FC.

## 4. Conclusions

In this study, seven biogenic AgNPs were obtained using the fungi species *A. tubingensis*, *Aspergillus* spp., *B. ochroleuca*, *C. pini-ponderosae*, *F. proliferatum*, *E. nigrum*, and *E. rostratum* isolated from the Brazilian biodiversity. Among them, this is the first report of *E. rostratum*, *F. proliferatum*, and *C. pini-ponderosae* application on the biosynthesis of AgNPs. The nanoparticles showed spherical morphology, with pH from 4.5 to 7.5, and size in the range from 43.4 to 120.6 nm (DLS) and from 21.8 to 35.8 nm (TEM). The functional groups from the biomolecules surrounding the AgNPs were analyzed by FTIR.

The AgNPs showed antifungal activity against clinical strains of *C. albicans*, *C. krusei*, *C. glabrata*, *C. parapsilosis*, *C. tropicalis*, and *C. guilliermondii*, common in hospital infections and against phytopathogens, responsible for serious damages in agricultural production. As expected, the results indicated that the physicochemical parameters of the AgNPs including the functional groups present on their surface interfere on their antifungal activity. Overall, the results indicate that there is no specificity of the AgNPs for yeasts or phytopathogens, which can be an advantage, increasing the possibility of application in different areas.

## Figures and Tables

**Table 1:** Physicochemical properties of the AgNP-Asp, AgNP-AT, AgNP-BO, AgNP-CCP, AgNP-EN, AgNP-ER, and AgNP-FP: size (nm) by DLS and TEM, polydispersity index (PDI), zeta potential, and pH values.

AgNPs	Size (DLS) *	Size (TEM) *	PDI	Zeta Potential	pH
AgNP-Asp	44.9 ± 4.1	33.3 ± 2.7	0.343	−1.04	6.0
AgNP-AT	43.4 ± 3.3	25.0 ± 6.5	0.080	−22.26	7.0
AgNP-BO	120.6 ± 3.5	21.8 ± 4.1	0.257	−2.19	7.5
AgNP-CPP	87.1 ± 3.4	35.8 ± 5.16	0.334	−1.59	5.0
AgNP-EN	71.2 ± 6.7	28.0 ± 6.31	0.404	−33.28	5.0
AgNP-ER	86.4 ± 6.4	22.1 ± 2.9	0.230	−14.40	4.5
AgNP-FP	59.6 ± 2.1	26.7 ± 5.3	0.199	−15.33	6.0

* Mean ± standard deviation (*n* = 6).

**Table 2:** Profile of vibrational frequencies (cm^−1^) and functional groups present in the AgNP-Asp, AgNP-AT, AgNP-BO, AgNP-CPP, AgNP-EN, AgNP-ER, and AgNP-FP. Data obtained using Fourier-transform infrared (FTIR) spectroscopy.

AgNPs	≈Vibrational Frequencies (cm^−1^)	Functional Groups
AgNP-Asp	825–860	Aromatic ring (2 adjacent H)
	1020–1170	C-O (ether)
	1320	C-N (aromatic)
	1560–1490	N-H
	1790	C=O of acyl chloride
	2925	Aliphatic C-H
	3200	O-H (chelate)
AgNP-AT	840–775	R_2_C=CHR (C-H out of plane)
	1165–1040	C-O (ether)
	1300	C-O (carboxyl)
	1590–1500 2927	C=C (aromatic) Aliphatic C-H
	3062	C-H (aromatic)
	3200	O-H (chelate)
AgNP-BO	825	Aromatic ring (2 adjacent H)
	1030–1018	C-O (ether)
	1350–1300	C-O (ester)
	1648	C=O (amides)
	2929–2900	Aliphatic C-H
	3400–3263	O-H (chelate)
AgNP-CPP	840–790	R_2_C=CHR (C-H out of plane)
	1160–1021	C-O (ether)
	1289	C-N (aromatic)
	1490	N-H
	1625	C=C (aromatic)
	2936	O-H (chelate)
	3330	Free NH (secondary amine)
AgNP-EN	945	RCH=CH_2_
	1190–1026	C-O (ethers)
	1336	SO_2_ (sulfone)
	1500	C=C (aromatic)
	2360	CO_2_
	2934	O-H (chelate)
	3343	Free NH (secondary amines)
AgNP-ER	820–798	R_2_C=CHR
	1170–1025	C-O (ether)
	1312	SO_2_ (sulfone)
	1493	N-H
	1621	C=C (aromatic)
	2936	Aliphatic C-H
	3230	O-H (chelate)
AgNP-FP	835–820	Aromatic ring
	1168–1020	C-O (ether)
	1300	C-O (ester)
	1490	N-H
	1550	NH_2_
	1630	C=O (amides)
	1790	C=O (acyl chloride)
	2925	C-H (aliphatic)
	3055	C-H (alkene)
	3200	O-H (chelate)

**Table 3:** Antifungal activity of the AgNP-Asp, AgNP-AT, AgNP-BO, AgNP-CPP, AgNP-EN, AgNP-ER, and AgNP-FP by minimal inhibitory concentration (MIC—µM) against an ATCC strain and clinical species of *Candida* sp.

Yeasts	AgNP-Asp	AgNP-AT	AgNP-BO	AgNP-CPP	AgNP-EN	AgNP-ER	AgNP-FP	AMB
*C. albicans* ATCC 36802	2.5	20	5	>40	1.25	20	10	32
*C. albicans* IOC 4556	10	40	20	>40	20	20	20	>32
*C. albicans* IOC 4525	2.5	20	20	>40	10	20	20	>32
*C. albicans* IOC 4558	10	40	20	>40	20	10	10	>32
*C. glabrata* IOC 4565	5	5	2.5	1.25	2.5	5	>40	>32
*C. krusei* IOC 4559	1.25	2.5	1.25	1.25	1.25	5	1.25	>32
*C. guillermondii* IOC 4557	1.25	2.5	1.25	1.25	1.25	2.5	1.25	32
*C. parapsilosis* IOC 4564	40	20	10	2.5	10	10	>40	>32
*C. tropicalis* IOC 4560	>40	20	20	5	20	10	5	>32

MIC = minimal inhibitory concentration refers to at least 90% of the yeast growth inhibition. AMB= amphotericin B; ATCC = American Type Culture Collection, IOC = Instituto Oswaldo Cruz Collection.

**Table 4:** Antifungal activity of the AgNP-Asp, AgNP-AT, AgNP-BO, AgNP-CPP, AgNP-EN, AgNP-ER, and AgNP-FP by minimal inhibitory concentration (MIC—µM) on the phytopathogens *C. lunata*, *F. sacchari*, *F. subglutinans*, *F. oxysporum* (WLA-FP07), *F. oxysporum* (WLA-FP25), and *F. verticillioides*.

Phytopathogens	AgNP-Asp		AgNP-AT		AgNP-BO		AgNP-CPP		AgNP-EN		AgNP-FP		AgNP-ER		AMB	
	MIC	FC	MIC	FC	MIC	FC	MIC	FC	MIC	FC	MIC	FC	MIC	FC	MIC	FC
*C. lunata*	8	8	4	4	16	16	4	4	4	4	4	4	4	4	4	4
*F. sacchari*	>250	>250	>250	>250	>250	>250	>250	>250	>250	>250	>250	>250	>250	>250	16	16
*F. subglutinans*	60	60	60	60	>250	>250	120	120	120	120	60	60	120	120	16	16
*F. oxysporum* (WLA-FP07)	120	120	120	120	>250	>250	250	250	120	120	120	120	120	120	>32	>32
*F. oxysporum* (WLA-FP25)	30	30	30	30	30	30	120	120	60	60	30	30	30	30	16	16
*F. verticillioides*	16	16	8	8	16	16	8	8	60	60	16	16	8	8	16	16

MIC = minimal inhibitory concentration refers to at least 90% of the fungi growth inhibition; AMB = amphotericin B; FC = fungicide concentration.

## Data Availability

Not applicable.
